# Anchor-Based Meniscal Ramp Repair

**DOI:** 10.1016/j.eats.2023.09.021

**Published:** 2024-01-01

**Authors:** Elizabeth Marks Benson, Audria Wood, Chandler Harris, Patrick Smith, John Xerogeanes, Aaron Casp, Amit Momaya

**Affiliations:** a Department of Orthopaedics, University of Alabama at Birmingham, Birmingham, Alabama; b Department of Orthopaedic Surgery, University of Missouri, Columbia; Columbia Orthopaedic Group, Columbia, Missouri; c Department of Orthopaedic Surgery, Division of Sports Medicine, Emory University, Atlanta, Georgia, U.S.A.

## Abstract

Ramp lesions of the medial meniscus are underdiagnosed because of difficulty in visualizing via magnetic resonance imaging and during arthroscopy. They most often occur simultaneously with anterior cruciate ligament (ACL) injury but may also be associated with posterior plateau contusions, steeper medial tibial plateau slope, and excess varus alignment. Upwards of 24% of ACL reconstructions have concomitant ramp lesions. Failure to repair the ramp lesion is associated with increased rotational laxity, tibial translocation, persistent pivot shift, and poorer outcomes after ACL reconstruction. The purpose of this article is to describe an all-suture anchor-based repair of a meniscal ramp lesion, which confers several advantages over traditional repair techniques.

A ramp lesion is generally described as a longitudinal tear within the posterior medial meniscocapsular junction.^1^ These lesions have garnered more attention over the last several years because of their association with continued knee dysfunction after anterior cruciate ligament (ACL) reconstruction. Originally discussed in the 1980s, these lesions were underdiagnosed because of difficulty visualizing the tear with magnetic resonance imaging or during arthroscopy.

Ramp lesions are most common during traumatic, acute knee ligament injuries or in chronic ACL-deficient knees.^2^ Ramp lesions are estimated to be present in upwards of 24% of patients undergoing ACL reconstruction.^1^^,^^3^^,^^4^ In acute injuries, men and patients younger than 30 are more likely to have simultaneous ACL and ramp lesions.^2^^,^^3^ Additional risk factors for ramp lesions include posterior medial tibial plateau contusions, steeper medial tibial and meniscus slopes, and varus knee alignment greater than 3°.^2^^,^^3^

Although controversial, stable lesions may heal without surgical intervention. However, many unstable lesions go undiagnosed; these have been associated with poor outcomes, such as increased degree of rotational laxity, tibial translocation, and persistent pivot shifts.^2^^,^^4^ A systematic review shows 7% to 8% of repairs fail, specifically 21% fail using an all-inside suture device.^1^ Excessive knee motion leads to biomechanical instability that may place greater loads on the ACL graft.

Multiple techniques have been described to address ramp lesions with most of them focused on a soft tissue repair between the meniscus and capsular tissue. We propose a technique that uses a tibial-based all-suture knotless anchor to repair the ramp lesion.

## Surgical Technique

Supplemental instructions for the key stages of the surgical technique are found in Video 1.

### Positioning

**Fig 1:**
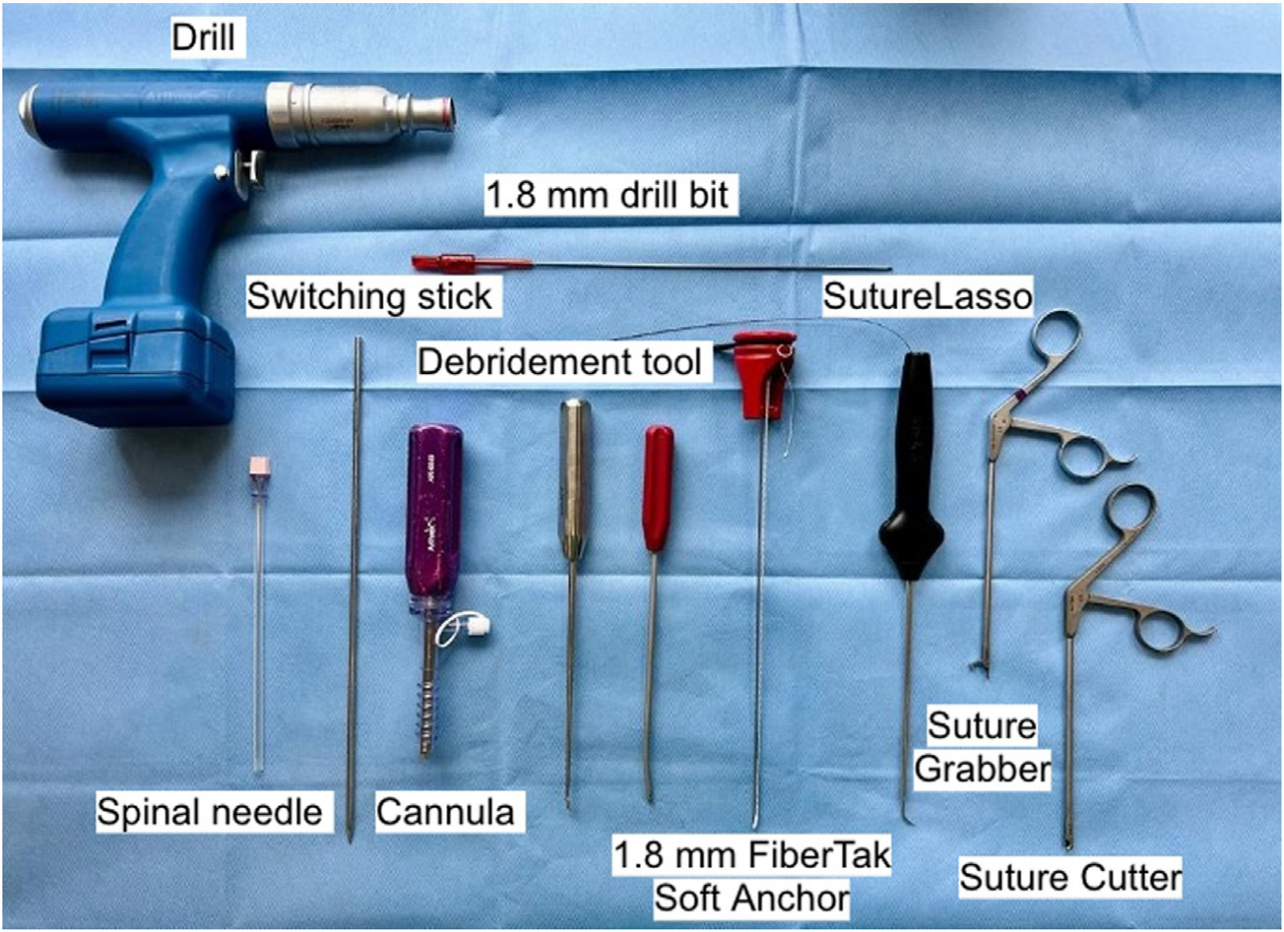
Required instrumentation specific to this technique. Shown equipment includes drill and 1.8 mm drill bit (top row from left to right); spinal needle, switching stick, cannula, debridement tool, 1.8 mm FiberTak soft anchors (Arthrex, Naples, FL), SutureLasso (Naples), suture grabber, and suture cutter (bottom row from left to right).

The operative knee should be flexed to approximately 90°. An L-bar may be used on the operative table to support the foot or flexed off the lateral edge of the table. A high thigh tourniquet is used. Positioning should provide access for anterolateral, anteromedial, and posteromedial portals with required instrumentation set up on the back table (Fig 1).

### Diagnostics

**Fig 2:**
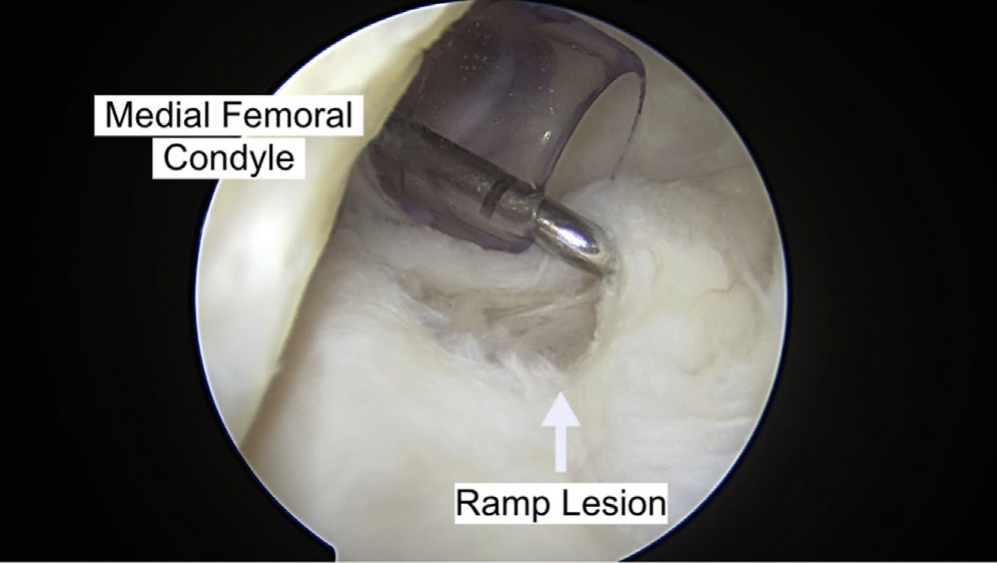
The patient remains supine with the knee flexed to approximately 90°. A probe is used in the posteromedial compartment to evaluate the ramp lesion. Probing the ramp lesion can help determine size, stability, and location of the lesion.

Magnetic resonance imaging and physical examination findings, although not definitive, can be indicative of the presence of ramp lesions. Diagnostic arthroscopy is initiated through the standard anterolateral and anteromedial portals. The medial compartment is assessed with the leg in slight valgus in extension. Often, the meniscal ramp lesion is not identified when viewing through the anteromedial compartment. The lateral compartment is evaluated with the leg in figure-four position. The meniscus and cartilage are probed. Any notch work, including ACL debridement or notchplasty, is completed. With the camera in the anterolateral portal, a modified Gillquist portal is achieved by placing the arthroscope between the medial wall of the intercondylar notch and the posterior cruciate ligament. This provides visualization of the posteromedial joint space. A 30° or 70° arthroscope may be used depending on visualization. The posterior compartment is inspected with careful attention to the meniscocapsular junction to identify tear(s), location, and size (Fig 2). Manual pressure on the posteromedial aspect of the knee can aid in bringing more of the meniscocapsular interface into view.

### Repair

**Fig 3:**
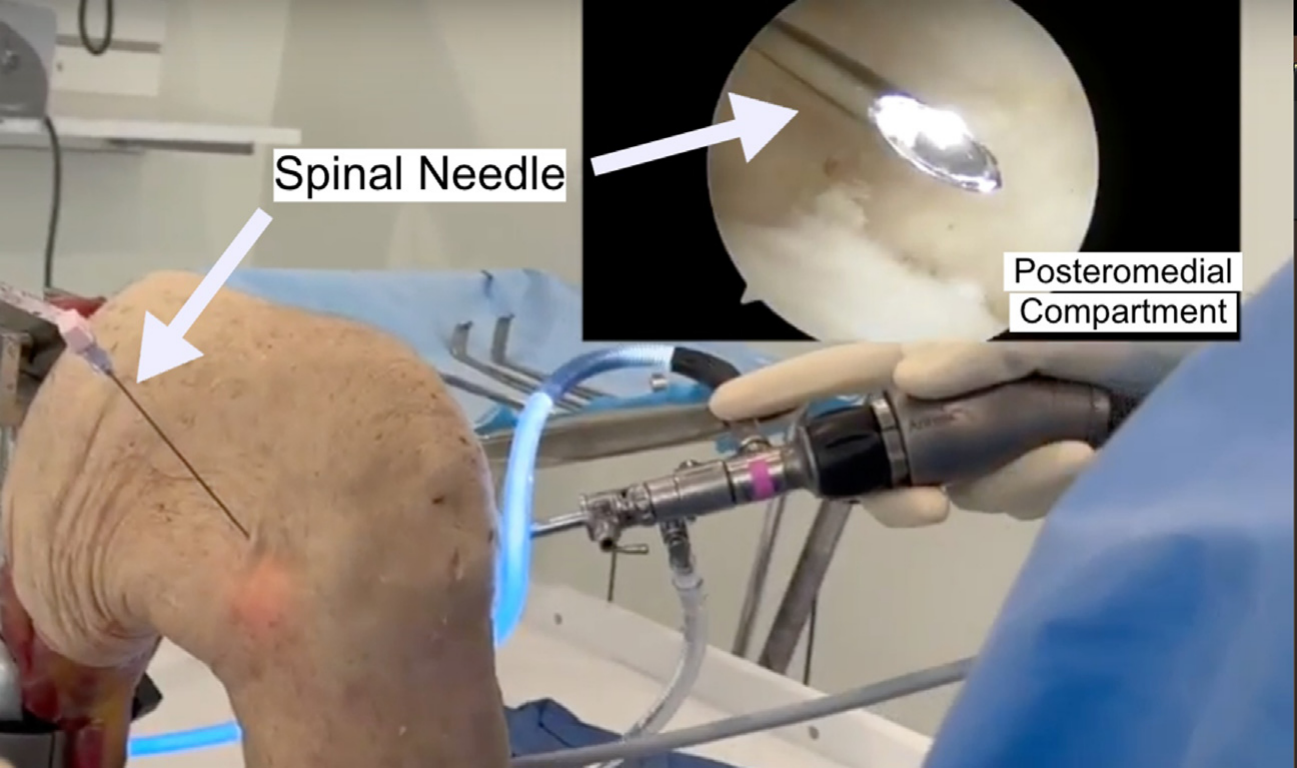
A cadaveric limb is pictured; a normal operating setting would position the patient supine with the knee supported by an L-bar at approximately 90°. A spinal needle is inserted to localize the posteromedial portal under direct visualization to ensure correct trajectory and placement to prevent injury to the saphenous vein and nerve.

**Fig 4:**
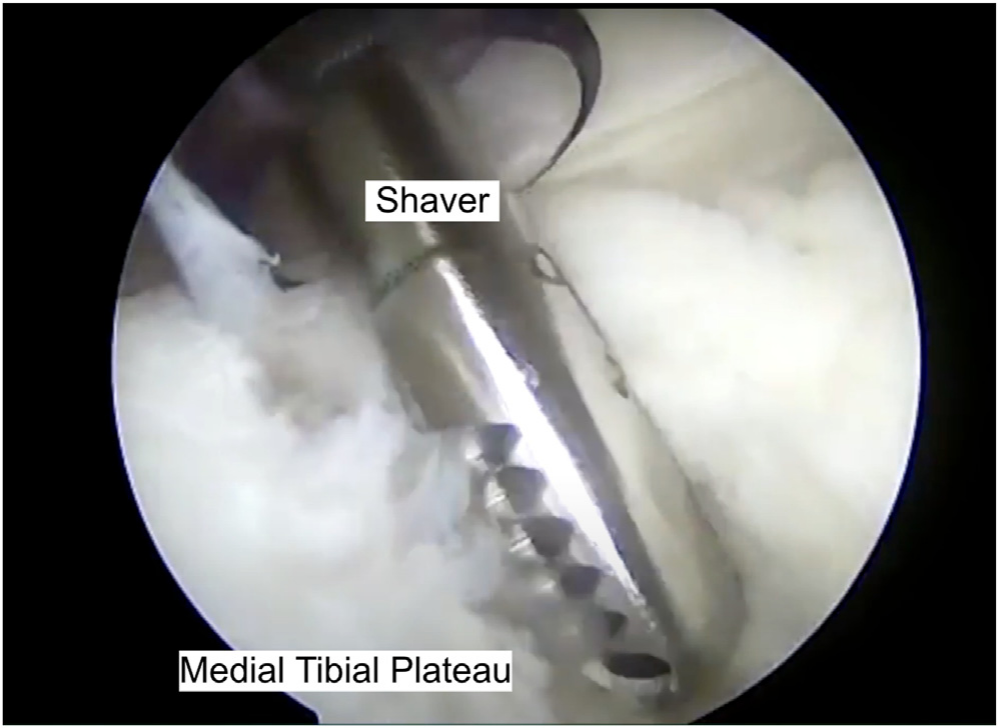
A shaver is inserted into the posteromedial compartment and used to biologically prepare the medial tibial plateau to facilitate healing after the ramp repair.

A posteromedial portal is created under direct visualization and transillumination to prevent injury to the saphenous vein and nerve. A spinal needle is first used to assess trajectory (Fig 3). Next, a small stab incision, using an 11-blade, is created along the posteromedial knee, followed by placement of a switching stick into the posteromedial capsule until visualized inside the joint, and a working cannula is placed. The tear is biologically prepared with a rasp and spinal needle to improve healing (Fig 4).

**Fig 5:**
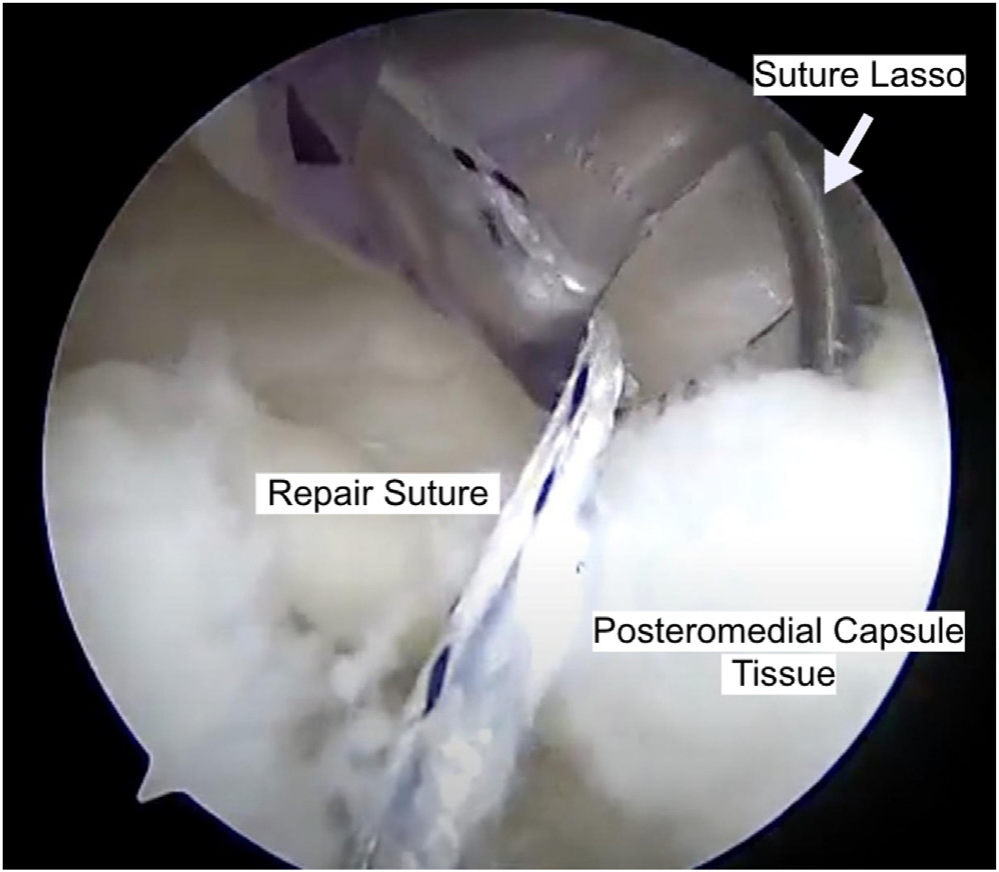
The all-suture knotless anchor has been placed along the posteromedial tibial plateau. The SutureLasso (Arthrex, Naples, FL) is used to capture the posteromedial capsule tissue. The repair stitch is then passed through this lasso. Once passed, the repair stitch is then placed into the soft tissue passing suture and tensioned down.

**Fig 6:**
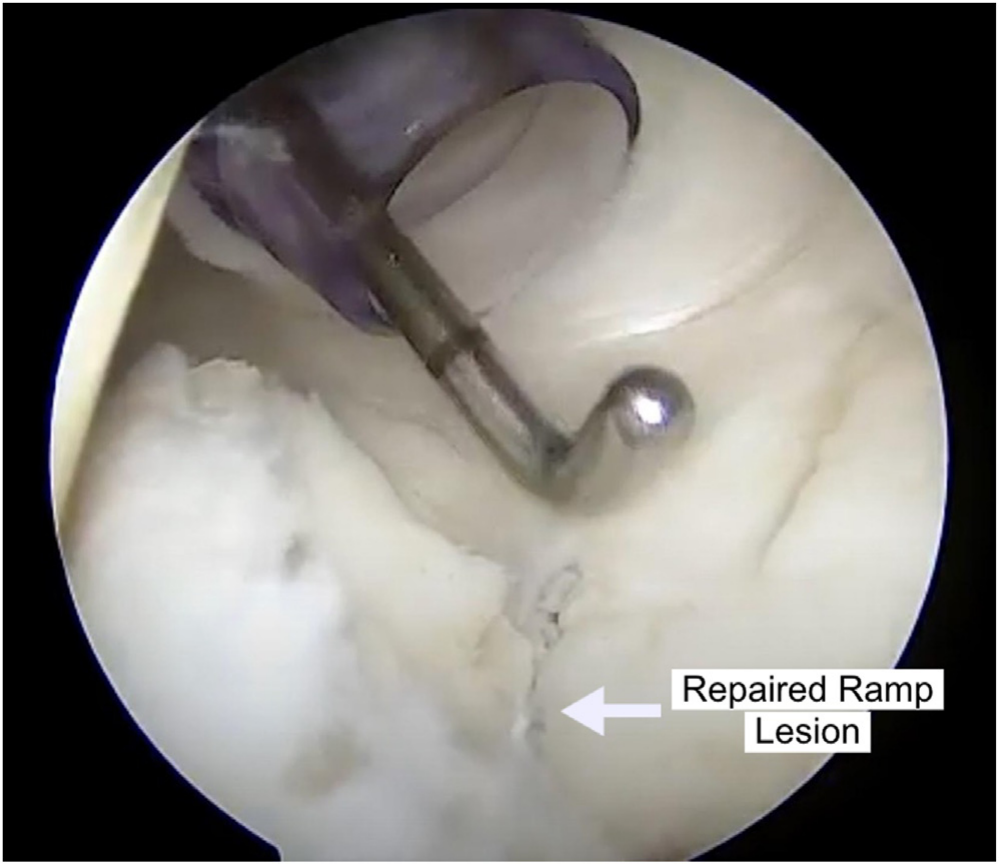
The completed repair is shown after the suture is cut flush, and a probe is used to assess the stability of the repair. After completion, attention is directed toward concomitant pathology of the knee.

Through the posteromedial working cannula, a curved anchor guide is placed through the meniscocapsular tear onto the posteromedial tibial plateau just adjacent to the meniscus side of the tear. Once drilled, a 1.8 mm knotless, all-suture FiberTak soft anchor (Arthrex, Naples, FL) is placed and tensioned to ensure anchor deployment (Fig 5). A SutureLasso (Arthrex) is used to grab the posteromedial joint capsule and exit within the tear on the capsular side. In this technique, the suture lasso is passed only through the posteromedial joint capsule and not the meniscus. This is advantageous in situations where the posteromedial femoral condyle blocks appropriate lasso passage through the meniscus. A variation of this technique may involve passing the suture through the meniscus also. The shuttling wire from the SutureLasso is then retrieved through the posteromedial cannula. The repair suture from the anchor is then passed through the nitinol wire loop and then shuttled through the posteromedial joint capsule. The repair stitch is then passed through the knotless mechanism in the anchor using the shuttling stitch. At this point, the repair stitch is through the posteromedial capsular tissue and back into the anchor. The repair suture is tensioned to the desired amount. Additional tissue can be grasped through the loop in the repair suture to incorporate additional capsule in the repair. Use caution to not overtension the posteromedial capsule. Once desired tension is achieved, the repair suture is cut. The meniscal ramp lesion is re-evaluated with a probe to ensure stability (Fig 6). The remaining concomitant pathology of the knee is addressed as indicated.

### Postoperative Protocol

For an isolated ramp repair, patients typically can partially bear weight for 2 weeks, followed by progression to full weightbearing as tolerated thereafter. Patients may begin range of motion as tolerated immediately. Patients will initiate running at approximately 10 weeks, followed by return to sport-specific activities around 12 weeks. Regarding ramp lesions in the setting of an ACL pathology, the only modification to standard ACL rehabilitation protocol is protected weightbearing in the first 2 weeks. Otherwise, a typical ACL protocol can be followed.

## Discussion

This surgical technique describes the use of an all-suture knotless anchor to address meniscus ramp lesions. The justification for this technique is rooted in its ability to reapproximate the posteromedial capsule with a high-strength construct.

There are multiple techniques to consider when addressing meniscal ramp lesions including the suture hook repair,^5^ all-inside,^6^ and inside out techniques^7^—each with their own advantages and disadvantages as seen in Table 1.

**Table 1:** Technique Comparison Table

	Suture Hook^5^	All-Inside^5^^,^^6^	Inside-Out^7^	Anchor-Based Repair
Posteromedial Portal	Yes	Optional	Yes	Yes
Material	Suture shuttling device	All inside meniscal repair device	Suture shuttling device for vertical mattress suture	Suture anchor
Cost	$	$$$	$	$$
Fine-adjustment tension	Yes	No	No	Yes
Meniscus Violation	Yes	Yes	Yes	No

Although some techniques for repairing meniscus ramp lesions use the posteromedial portal, others have proposed ways to mitigate the need for it and rely exclusively on visualization from the intercondylar notch. However, the intercondylar notch provides a limited view of the posterior medial compartment and requires accessing the posterior meniscocapsular junction across the entirety of the chondral surface, so it may be associated with risks of iatrogenic cartilage damage. Increased visualization of the medial compartment can be achieved with pie-crusting of the medial collateral ligament; however, this may increase risk of injury to the saphenous vein, postoperative pain, and joint instability.^8^ The posteromedial portal provides the most comprehensive visualization of the posterior medial compartment, which is favorable for visualizing ramp lesions.^4^ Although a disadvantage of this portal is the risk of neurovascular injury, safe approaches have been described that minimize this risk, such as the use of transillumination when localizing the posteromedial portal.^4^

This technique involves the use of an all-suture anchor (ASA), which provides multiple advantages. ASAs have a high load-to-failure in biomechanical studies that have clinically translated to low retear rates, and the polyethylene composition has demonstrated a low complication rate, including low risk of infection.^9^ ASA have been used for fixation in other areas for soft tissue repair to bone and have shown favorable biomechanical results similar to the native state.^10^

Furthermore, an anchor-based repair confers multiple advantages over the traditional meniscus ramp repair techniques. The tensionable knotless mechanism allows for the surgeon to control suture tension and prevents loosening that may occur with knot-tying. This knotless construct also avoids any intra-articular knot-stack, which could irritate the tissues in the posteromedial space.^10^ This technique also has the theoretical advantage of enhanced healing because of the release of bone marrow elements at the site of the tear from the anchor drill-tunnel. This anchor-based repair may also provide some technical advantage; passing a suture shuttling device through the capsule and meniscus can often be technically difficult, because the posteromedial condyle can block the instrumentation. Our technique does not require the meniscus edge to be pierced but instead approximates the posteromedial capsular tissue back to the meniscus at its native location.

This technique is not without its potential disadvantages. If the bone quality is poor, the ASA may not deploy appropriately. This can be addressed with the substitution of a PEEK anchor device SutureTak (Arthrex) also with a knotless mechanism. There is also potential to overtension the repair, theoretically overtightening the posteromedial capsule. This can be mitigated by placing the knee in partial extension when performing final repair-suture tightening. In addition, in this technique, the capsule is not anatomically repaired back to the meniscus but rather approximated by anchoring it to the posterior tibia. Further studies are needed to determine if this affects stability. Using an anchor-based repair may also be more costly than a suture-only repair construct. A summary of advantages versus disadvantages and pearls versus pitfalls may be found in Table 2 and Table 3, respectively. In conclusion, we present a technique that uses a tibial-based knotless all-suture anchor repair to address meniscal ramp lesions.

**Table 2:** Advantages and Disadvantages of Anchor-based Ramp Repair

Advantages
High strength of repair
Direct visualization for assessment and repair
Ability to fine-tune the tension and re-tension
Generalizable to all classifications of ramp lesions
Enhanced biology because of release of bone marrow elements from drilling
Disadvantages
Increased cost because of anchor
Possible drill-path convergence in concomitant procedures
Requires moderate technical skill
Risk to saphenous structures
Requires assistance for drilling and anchor placement
Risk of anchor pullout in cases of poor bone quality
Does not anatomically repair capsule back to meniscus but rather approximates it

**Table 3:** Pearls and Pitfalls of Anchor-Based Ramp Repair

Pearls
Assess from standard anterior portals, trans notch view, and posteromedial portal
Use a needle and transillumination for posteromedial portal placement
Have complete visualization of the ramp lesion
Biologically prepare the lesion
Gentle mallet taps on the guide before drilling to set the anchor location and avoid slippage of the guide
Pull back on anchor to confirm appropriate deployment and recognize potential pull-out
Tension and possible re-tension appropriately
Pitfalls
Potential neurovascular injury in posterior knee
Be cautious of this technique if concomitant meniscal root tear because of potential tunnel convergence
Avoid overtensioning the repair

## Disclosure

The authors report the following potential conflicts of interest or sources of funding: A.M. reports board membership/owner/officer/committee appointments for *Arthroscopy*; is a paid consult or employee for Arthrex and Fidia Pharma USA; and is an unpaid consultant for Miach Orthopaedics and Reparel. P.S. reports board membership/owner/officer/committee appointments for *Arthroscopy,* the American Orthopaedic Society for Sports Medicine, and the *Journal of Knee Surgery*; royalties from Arthrex; Speakers bureau/paid presentations from Arthrex; research or institutional support from Arthrex; stock or stock options from Spinal Simplicity; is a paid consultant or employee for Arthrex. J.X. reports royalties from Arthrex; and stock or stock options from My-Eye; is a paid consultant or employee for Arthrex and Trice Medical. A.C. is a paid consultant or employee for Arthrex. Full ICMJE author disclosure forms are available for this article online, as supplementary material.

## Supplementary Data

ICMJE author disclosure forms

Video 1 Meniscal ramp lesion repair utilizing an anchor-based technique. With the patient supine and the knee supported by an L-bar at approximately 90° flexion, the posterior compartment of the medial meniscus is inspected then the ramp lesion is prepared. An all-suture anchor is placed, and the sutures are used to repair the capsule.
